# Heterologous and High Production of Ergothioneine in *Bacillus licheniformis* by Using Genes from Anaerobic Bacteria

**DOI:** 10.3390/metabo15010045

**Published:** 2025-01-12

**Authors:** Zhe Liu, Fengxu Xiao, Yupeng Zhang, Jiawei Lu, Youran Li, Guiyang Shi

**Affiliations:** 1 School of Biotechnology, Key Laboratory of Carbohydrate Chemistry, Biotechnology of Ministry of Education, Jiangnan University, Wuxi 214122, China; 6220210060@stu.jiangnan.edu.cn (Z.L.); 8202306022@jiangnan.edu.cn (F.X.); 8202407007@jiangnan.edu.cn (Y.Z.); lujiawei@stu.jiangnan.edu.cn (J.L.); 2 National Engineering Research Center for Cereal Fermentation and Food Biomanufacturing, Jiangnan University, 1800 Lihu Avenue, Wuxi 214122, China; 3 Jiangsu Provincial Engineering Research Center for Bioactive Product Processing, Jiangnan University, Wuxi 214122, China

**Keywords:** ergothioneine, whole-cell transformation, *Bacillus licheniformis*, amino acids, methyltransferases, sulfur transferases

## Abstract

Purpose: This study aimed to utilize genetically engineered *Bacillus licheniformis* for the production of ergothioneine (EGT). Given the value of EGT and the application of *Bacillus licheniformis* in enzyme preparation production, we cloned the key enzymes (EanA and EanB) from *Chlorbium limicola*. Through gene alignment, new ergothioneine synthase genes (EanAN and EanBN) were identified and then expressed in *Bacillus licheniformis* to construct strains. Additionally, we investigated the factors influencing the yield of EGT and made a comparison with *Escherichia coli*. Methods: The relevant genes were cloned and transferred into *Bacillus licheniformis*. Fermentation experiments were conducted under different conditions for yield analysis, and the stability of this bacterium was also evaluated simultaneously. Results: The constructed strains were capable of producing EGT. Specifically, the yield of the EanANBN strain reached (643.8 ± 135) mg/L, and its stability was suitable for continuous production. Conclusions: Genetically engineered *Bacillus licheniformis* demonstrates potential in the industrial-scale production of EGT. Compared with *Escherichia coli*, it has advantages, thus opening up new possibilities for the application and market supply of EGT.

## 1. Introduction

Ergothioneine (EGT) is a natural amino acid derivative, a thiol compound derived from histidine [1]. Studies have shown that EGT possesses strong antioxidant properties and various biological functions, including UV absorption, color protection, cellular energy regulation, anti-inflammatory effects, antidepressant activity, promotion of neuron differentiation, immune regulation, and anti-aging [2]. These unique biological functions make it highly promising for applications in functional foods as a new and non-toxic natural food preservative [3]. Secondly, in the cosmetics industry, EGT is proven to be a safe, non-acne-inducing and skin-friendly active ingredient [4]. In addition, studies have shown that EGT plays a positive role in the prevention of eye diseases and the treatment of cardiovascular disease, cancer, diabetes, neurodegenerative diseases, and preeclampsia [5].

Despite the broad application prospects of EGT, its market price remains quite high. Therefore, more economical and efficient production methods are needed to provide more possibilities for the large-scale production of EGT. EGT production methods are mainly divided into three categories: biological extraction [6], chemical synthesis [7], and biosynthesis [8]. Biological extraction involves extracting EGT from various edible fungi, but this method faces challenges such as low EGT content and high impurity levels in the raw materials. Currently, some manufacturers use chemical synthesis and biological extraction to produce EGT, with chemical synthesis still being the primary source of EGT. However, chemical synthesis methods have the problem of expensive raw materials, high reaction temperatures, or the need for multiple chromatography purification, resulting in a waste of resources and increased costs. With the use of many hazardous chemical reagents, it is necessary to treat waste liquid and waste in multiple steps to avoid environmental pollution [9].

Although the EGT biosynthetic pathway in *C. limicola* has been elucidated [10,11,12,13], research on this pathway is still limited, possibly due to a lack of suitable expression hosts. At present, the two enzymes EanA and EanB are recombinant expressed in *Escherichia coli*, cultured in a shaking medium, and induced with IPTG. Then, the proteins are collected and purified, and cysteine desulfurase (IscS) from *E. coli* is added to provide a sulfur source for EanB. This greatly increases the workload and complexity of the experiment. Increasing research on this pathway could uncover more new EGT synthases and expand the range of host cells, thereby meeting the growing market demand for ergothioneine. As a facultative anaerobe with abundant enzyme production capabilities [14], *Bacillus licheniformis* can serve as a chassis strain for ergothioneine production.

This study utilized *Bacillus licheniformis* (ATCC 9945a) as the starting strain to construct the first engineered strain synthesizing ergothioneine via the *Chlorobium limicola* pathway. Based on this, we optimized ergothioneine production using whole-cell catalysis, exploring the effects of different temperatures and the initial pH on ergothioneine production, thereby further enhancing the engineered strain’s ability to synthesize ergothioneine. Finally, to investigate the impact of substrate amino acid concentration on ergothioneine production, we measured extracellular amino acids and optimized the combination and concentration of amino acids.

## 2. Materials and Methods

### 2.1. Strains and Plasmids

*B. licheniformis* (ATCC 9945a), *Escherichia coli* JM109, and plasmid pHY300-PLK were preserved in our laboratory. Antibiotics were used at final concentrations: ampicillin (Amp) 100 μg·mL^−1^ and tetracycline (Tet) 20 μg·mL^−1^. *B. licheniformis* (ATCC 9945a) served as the expression strain for ergothioneine synthesis. The strains and plasmids used in this study are listed in Table 1.

### 2.2. Reagents

Ergothioneine standard samples were purchased from Shanghai Macklin Biochemical Co., Ltd (Shanghai, China).; methionine was purchased from Shanghai Macklin Biochemical Co., Ltd (Shanghai, China).; cysteine and histidine were purchased from Aladdin Reagent Co., Ltd (Shanghai, China).; ampicillin and tetracycline were purchased from Sigma-Aldrich (St.Louis, MO, USA); and all other reagents were analytical grade from domestic suppliers.

### 2.3. Media

LB medium: 5 g/L yeast extract, 10 g/L tryptone, 10 g/L NaCl; for LB solid medium, add 1.5% (*w*/*v*) agar. Whole-cell catalyst preparation medium: 50 g/L liquid nitrogen source, 1.36 g/L KH_2_PO_4_, 6.96 g/L K_2_HPO_4_, 10 g/L (NH_4_)_2_HPO_4_, 10 g/L yeast extract, 10 g/L tryptone, 2 g/L corn steep liquor powder, 1 g/L NaCl, 100 g/L glucose. Electroporation medium: 0.5 mol·L^−1^ sorbitol, 0.5 mol·L^−1^ mannitol, 10% glycerol. Transformation buffer: 1.5 g/L MgSO_4_·7H_2_O, 1.36 g/L KH_2_PO_4_, 6.96 g/L K_2_HPO_4_, with additional glucose and various concentrations of amino acid stock solutions (filtered for sterility). All prepared media included tetracycline at a final concentration of 20 μg·mL^−1^ for the fermentation culture of the strains.

### 2.4. Construction of Recombinant Plasmids

The genes EanA, EanB, EanAN, and EanBN were synthesized by GenScript (Suzhou) Co., Ltd(Suzhou, China)., with EanA and EanB originating from *C. limicola*, EanAN from *Bacteroidales bacterium*, and EanBN from *Anaerobacillus alkalidiazotrophicus*. Plasmids were constructed using one-step cloning or enzyme digestion, verified by colony PCR and sequencing. The recombinant plasmids were transformed into host strains using the *B. licheniformis* transformation method [15]. Transformants were selected on tetracycline-resistant plates (20 μg·mL^−1^) and verified by PCR using pHY300-PLK universal primers. Correct transformants were further verified by restriction enzyme digestion and electrophoresis and named EanAB and EanANBN.

### 2.5. Shake Flask Fermentation

During bottle shaking culture, a single colony of ergothioneine recombinant bacteria on the tetracycline plate is inoculated in a 1 mL LB liquid medium, incubated at 37 °C for 16 h, and used to prepare the preculture. For the main culture, transfer 1 mL of preculture to a 30 mL fermentation medium in a 250 mL flask and rotate and incubate at a speed of 250 rpm per minute at 37 °C.

### 2.6. Whole-Cell Transformation

Shake flask culture: the seeds were prepared using the method of Shake Flask Fermentation. The activated bacterial solution was connected to the whole-cell catalyst medium prepared with a 3% inoculation amount for culture. The bacteria were collected after fermentation and culture at 37 °C and 250 rpm for 48 h, and the fermentation broth was centrifuged at 12,000 rpm and 4 °C for 15 min to discard the supernatant and collect the bacteria. The collected cells were washed twice with pre-chilled 0.9% NaCl solution, and after the washing was completed, the supernatant was removed by centrifugation under the same conditions. Then, the cells were resuspended in 0.9% NaCl solution to form the desired cell suspension at the desired cell concentration, and the proportion of the pre-sterilized transformation buffer and tetracycline at a final mass concentration of 20 μg·mL^−1^ tetracycline were added.

### 2.7. Sample Processing for Ergothioneine

The fermentation broth was centrifuged at 12,000 rpm for 8 min, the supernatant was collected, and an equal volume of 10% trichloroacetic acid solution was added to precipitate proteins at 4 °C. After centrifugation, the supernatant was diluted to an appropriate concentration and filtered through a 0.22 μm aqueous membrane for analysis.

### 2.8. Quantitative Determination of Ergothioneine

The concentration of ergothioneine was determined using high-performance liquid chromatography (HPLC). The Thermo HPLC system with a C8 column (ZORBAX RX-C8, Analytical 250 mm × 4.6 mm, 5-Micron) and UV detector was used. The detection wavelength was set at 257 nm, with a mobile phase consisting of 5% methanol in water, and pure methanol as the organic phase. The flow rate was set at 0.7 mL/min, the injection volume was 10 μL, and each sample analysis lasted for 20 min with the column temperature maintained at 30 °C. The standard curve for ergothioneine: Standard samples with concentrations of 5, 10, 20, 50, 100, and 200 mg/L were prepared. The peak areas corresponding to each concentration were measured by HPLC, and a standard curve was plotted with the concentration of ergothioneine on the *x*-axis and the corresponding peak area on the *y*-axis.

### 2.9. Other Detection Methods (OD600, Amino Acids)

The growth of the bacterial cells was periodically sampled, diluted to appropriate concentrations, and measured for OD_600_ using a UV spectrophotometer. Glucose concentration was measured using the DNS method to determine residual sugar concentration [16]. Amino acid concentration determination extracellular amino acid concentrations were measured using high-performance liquid chromatography (HPLC). The Thermo HPLC system with a C18 column and UV detector was used, with a detection wavelength of 360 nm. Mobile phase A consisted of 50% acetonitrile, and mobile phase B consisted of 8.2 g anhydrous sodium acetate dissolved in 1800 mL water, 200 mL acetonitrile, 2 mL triethylamine, and 2 mL of a 1 g/L EDTA solution. Pure methanol was used as the organic phase. The injection flow rate was 0.8 mL/min, the injection volume was 10 μL, and the analysis time for each sample was 30 min, with the column temperature maintained at 30 °C throughout.

## 3. Results and Discussion

### 3.1. Biosynthetic Pathways of Ergothioneine and Strategies for Yield Optimization

Ergothionein biosynthesis can be divided into two approaches: one of a kind, producing EGT through submerged fermentation using naturally EGT-synthesizing microorganisms [17], such as naturally edible mushrooms, and another kind, constructing engineered strains. One approach is to select strains that inherently have EGT production capabilities, such as *Methylobacterium* [18], and directly enhance the key enzymes in the EGT synthesis pathway to boost EGT synthesis. On the other hand, strains with clear genetic backgrounds, convenient genetic modification, and superior metabolic abilities can be selected as chassis bacteria to recombine various bacterial and fungal EGT synthases and express them in excellent host cells. Additionally, metabolic regulation and fermentation optimization can effectively improve ergothioneine production efficiency, making it the optimal choice for the industrial production of ergothioneine [17].

The synthesis of EGT using recombinant microorganisms is mainly achieved by exploring natural biosynthetic pathways, discovering key enzymes, and efficiently expressing them in specific hosts. Nowadays, many microbial EGT biosynthetic pathways have been elucidated: bacterial pathways such as the EgtABCDE gene cluster (encoding the five EGT synthases EgtA, EgtB, EgtC, EgtD, and EgtE) in *Mycobacterium smegmatis* [19], fungal pathways like the Egt1 and Egt2 genes in *Neurospora crassa* [20], and anaerobic bacterial pathways such as the EanA and EanB genes in *Chlorbium limicola* [10], all of which use L-His, L-Cys, and L-Met as precursor substances. Current research mainly focuses on the first two biosynthetic pathways, emphasizing the screening and heterologous expression of synthases, optimization of precursor addition, or overexpression of genes related to precursor synthesis [21]. For instance, Osawa [8] et al. achieved an EGT yield of 24 mg/L by expressing the EgtBCDE gene cluster from *M. smegmatis* in *E. coli* combined with optimization of the medium and precursor supply. Van der Hoek [22] et al. optimized various combinations of fungal and bacterial EGT synthases, expressing *N. crassa*-derived Egt1 and *C. purpurea*-derived Egt2 in a *S. cerevisiae* strain, and achieved an EGT yield of 598 mg/L after 84 h of fed-batch fermentation by optimizing precursor amino acid supply. Zhang [23] et al. heterologously expressed EgtE in *E. coli*, engineered EgtD and bifunctional enzyme NcEgt1 (TNcEgt1) through protein engineering, and obtained an EGT yield of 5.4 g/L after 96 h of fed-batch fermentation with amino acid supplementation, which is the highest level of EGT production using *E. coli* to date (Table 2). These findings indicate that screening for EGT synthases and optimizing precursor supply are essential for enhancing EGT yield.

**Figure 1:**
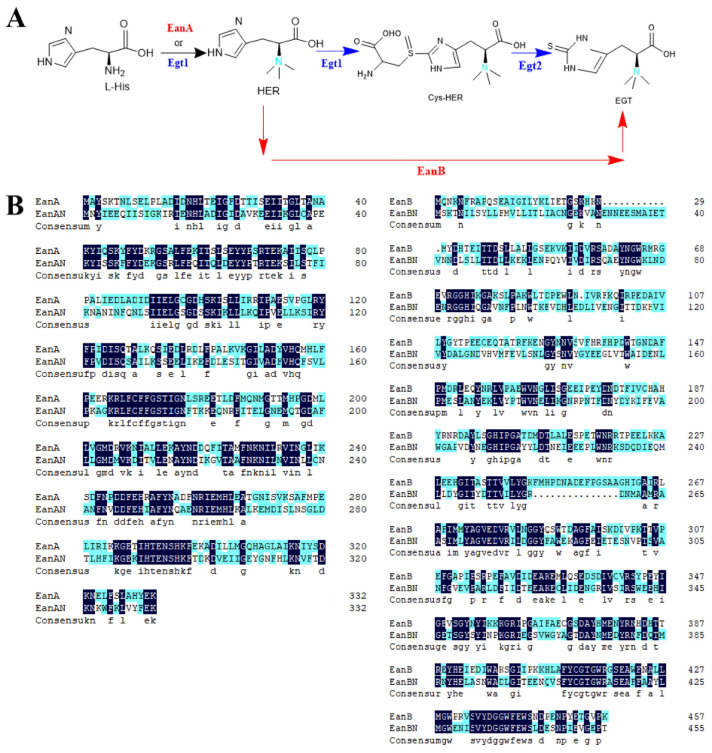
*N. crassa* pathway versus comparison of the *C. limicola* pathway. Blue represents biosynthetic pathways of EGT in *N. crassa* [Egt1: Bifunctional enzymes (SAM-dependent histidine methyltransferase and mononuclear non-heme iron enzyme); Egt2: PLP-mediated C-S lyase]. Red represents biosynthetic pathways of EGT in *C limicola* [EanA: Methyltransferase; EanB: rhodanese-like sulfur transferase] (**A**) and amino acid sequence alignment of methyltransferases (EanA and EanAN) and sulfurtransferases (EanB and EanBN) from two different sources [Dark blue indicates sequence consistency] (**B**).

The *M. smegmatis* pathway requires more enzymes for synthesis compared to the other two pathways, making it more complex with greater competition and inhibition, and also requires the involvement of L-glutamic acid [19]. The *N. crassa* pathway differs from the *C. limicola* pathway (FIG. 1). In the *N. crassa* pathway, Egt1 is a bifunctional enzyme that first catalyzes the conversion of histidine to HER (hercynine—an intermediate metabolite derived from histidine through the catalytic activity of methyltransferases), then catalyzes the synthesis of Cys-HER from HER and cysteine, followed by Egt2 catalyzing the conversion of Cys-HER to EGT. In contrast, the *C. limicola* pathway involves EanA catalyzing the conversion of histidine to HER, followed by EanB directly transferring a sulfur atom to the imidazole ring of HER under anaerobic conditions to produce EGT [24].

**Table 2:** Fermentation levels of genetically engineered strains for ergothioneine.

Strain	Key Strategy	Growth Conditions	Yield	Fermantation Period/h	Reference
*E. coli* E1-A1-thrA-serAc.g *	Histidine supply and methyl donor were optimized, and CaCl_2_ was added to improve cell membrane permeability	Shake flask	243.06 mg/L	108	[25]
*Escherichia coli*	egtB from *Methylobacterium* *pseudosasicola*, egtDE from *Methylobacterium strain*	Shake flask	657 mg/L	192	[18]
*S*. *cerevisiae*	egt1 of Neurospora crassa and egt2 of *C. purpurea*	Fed-batch	598 mg/L	84	[22]
*E. coli* BL21 (DE3)	expression of egtEMs gene; semi-rational design and random mutations of EgtD and TNcEgt1	Fed-batch	5.4 g/L	96	[23]
*B. subtilis* 168	The active methyl cycle (AMC) and tricarboxylic acid (TCA) cycles were reconstituted	Fed-batch	619.42 mg/L	120	[26]
*E. coli* BW25113	Overexpression of Tregt1 and Tregt2 genes	Fed-batch	4.34 g/L	143	[27]

* pCDFDuet-1 containing egtA, egt1 (From *Schizosaccharomyces pombe* genome amplification), thrA (From *E. Coli* BL21 (DE3) genome amplification) and serA^T410STOP^ (From *Corynebacterium glutamicum* ATCC 13032 genome amplification)

### 3.2. Mining of Ergothioneine Synthase Genes

**Figure 2:**
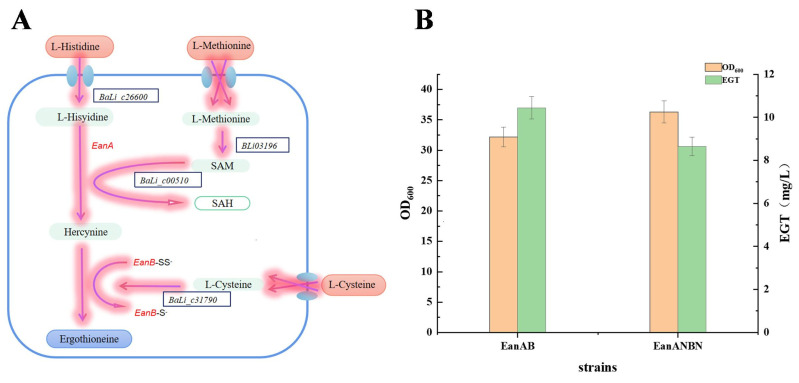
Ergothioneine biosynthesis pathway from *Chlorbium limicola* (**A**) and Ergothioneine production of two recombinant strains (**B**) are shown.

At present, there is no relevant study on the synthesis of ergothioneine by heterologous expression of the pathway of *Chlorobium limicola*. The discovery of ergothioneine synthase in *Chlorobium limicola* provides a new pathway and more possibilities for ergothioneine production. The production of ergothioneine by the anaerobic route could give more facultative anaerobes or anaerobes the opportunity to become chassis strains for ergothioneine production, and in industrial large-scale production, large-scale ventilation is not required to reduce costs. Therefore, this study utilized the NCBI database to identify and select the methyltransferase EanA and sulfurtransferase EanB from *Chlorobium limicola*. Further, to clone the EGT biosynthetic genes from *Chlorobium limicola*, the BLAST tool (https://blast.ncbi.nlm.nih.gov/Blast.cgi, accessed on 12 March 2023) was used to identify the corresponding sequence. Based on the gene sequences of *Chlorobium limicola* EanA (Clim_1148) and EanB (Clim_1149), we predicted that two hypothetical proteins, methyltransferase from *Bacteroidales bacterium* and sulfurtransferase from *Anaerobacillus alkalidiazotrophicus*, were involved in the EGT biosynthetic pathway and named EanAN and EanBN, respectively. DNAMAN was used for sequence alignment to analyze its conservatism. The amino acid sequence alignment results are shown in Figure 1. The amino acid sequences of EanA and EanAN were used as templates for BLAST online analysis, and the conserved domains of these two proteins belonged to the Superfamily. The mycobacterial members of this family are expressed from part of the ergothioneine biosynthetic gene cluster. EgtD is the histidine methyltransferase that transfers three methyl groups to the alpha-amino moiety of histidine. In the first stage of the production of this histidine betaine derivative that carries a thiol group attached to the C2 atom of an imidazole ring, the EanAN sequence was 333 bp, and it has 49.25% homology with EanA. Similarly, EanB and EanBN were analyzed, and the conserved domains of these two proteins also belonged to the Superfamily 3-mercaptopyruvate sulfurtransferase SseA, containing two rhodanese domains. The EanBN sequence was 456 bp and had 38.43% homology with EanB. These suggest that EanAN and EanBN may perform the same function as EanA and EanB. As shown in Figure 2, EanA catalyzes the conversion of histidine to HER, followed by EanB transferring a sulfur atom to the imidazole ring of HER under anaerobic conditions, producing ergothioneine [10].

### 3.3. Expression of Ergothioneine Synthase Genes

The *Bacillus licheniformis* compatible plasmid pHY300-PLK, containing the constitutive promoter P2, was selected as the vector. The ergothioneine synthesis pathway from *Chlorobium limicola* was constructed into the plasmid pHY-P2-EanAB and transformed into the host bacterium *B. licheniformis* (ATCC 9945a) via electroporation, resulting in the recombinant strain EanAB. Shake flask cultivation of strain EanAB followed by HPLC analysis showed that the recombinant strain could produce (12 ± 1.40) mg/L of ergothioneine (Figure 2). EanAN and EanBN were used to replace the corresponding catalytic steps to verify if EanAN and EanBN had the corresponding catalytic functions. By replacing EanA with EanAN and EanB with EanBN, the recombinant strain EanANBN was obtained using the same method. Shake flask cultivation and HPLC analysis showed that the recombinant strain EanANBN could synthesize ergothioneine with a yield of (9.8 ± 1.60) mg/L (Figure 2). This result indicates that both EanAN and EanBN can catalyze the corresponding reactions, successfully expressing the ergothioneine synthesis pathway from *Chlorobium limicola* in *Bacillus licheniformis*.

### 3.4. Whole-Cell Conversion for Ergothioneine Production

**Figure 3:**
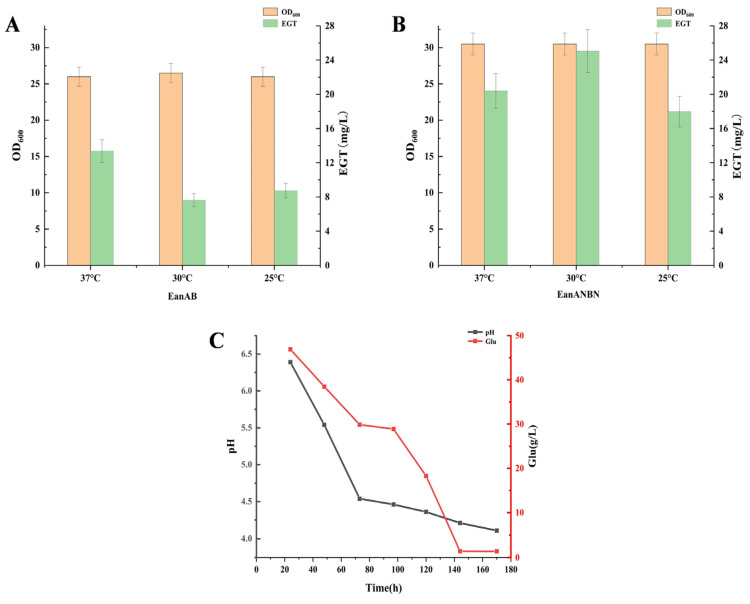
Ergothioneine is produced using the whole-cell transformation method. The yield of ergothioneine from recombinant bacteria EanAB at different temperatures (**A**), the yield of ergothioneine from recombinant bacteria EanANBN at different temperatures (**B**), and the detection of changes in pH and glucose concentration during the transformation process (**C**) are shown.

Although the biosynthetic pathway from *Chlorobium limicola* is clear, the catalytic rates of the identified EanA and EanB (kcat = 3.8 min^−1^ and 0.5 min^−1^) are much lower than those reported for the aerobic pathway enzymes EgtD and EgtB (kcat = 35 min^−1^ and 72 min^−1^) [10]. Therefore, EanAN and EanBN were used to replace EanA and EanB to verify that there are more alternative synthases to produce EGT. Whole-cell conversion was employed to increase the initial cell concentration for ergothioneine production. Since *Chlorobium limicola* lives in hypoxic water [10], three transformation temperatures (25 °C, 30 °C, and 37 °C) were chosen for whole-cell conversion to produce ergothioneine. Under the conversion condition of 25 degrees, the yield of EanAB was (8.9 ± 1.30) mg/L. At a conversion temperature of 30 degrees, the concentration of the product was not much different from that at 25 degrees, with (7.9 ± 1.30) mg/L. Under the conversion conditions of 37 degrees, the yield of EGT was the highest (13.5 ± 1.60) mg/L. EanANBN, a recombinant bacterium obtained by replacing methyl transferase and sulfur transferase, had a higher EGT yield than EanAB at the three temperatures. The EGT conversion yield at 25 degrees, 30 degrees, and 37 degrees was (18 ± 1.4) mg/L, (25.8 ± 1.5) mg/L, and (20.4 ± 1.3) mg/L, respectively (Figure 3).

For whole-cell conversion, temperature and pH are crucial factors affecting the conversion process [28]. An optimal transformation temperature is vital for maintaining high enzyme activity, while the pH environment during conversion affects intracellular enzyme activity and stability. Different pH levels can alter cell metabolism, membrane permeability, and protein expression, further influencing the accumulation of target products [29]. Therefore, optimizing the initial pH for whole-cell conversion is necessary to determine the optimal pH. It was observed that the pH decreased significantly during conversion (Figure 3), suggesting that the pH might influence the expression of ergothioneine-related synthetic enzymes. Thus, subsequent investigations focused on different initial pH values and temperatures.

**Figure 4:**
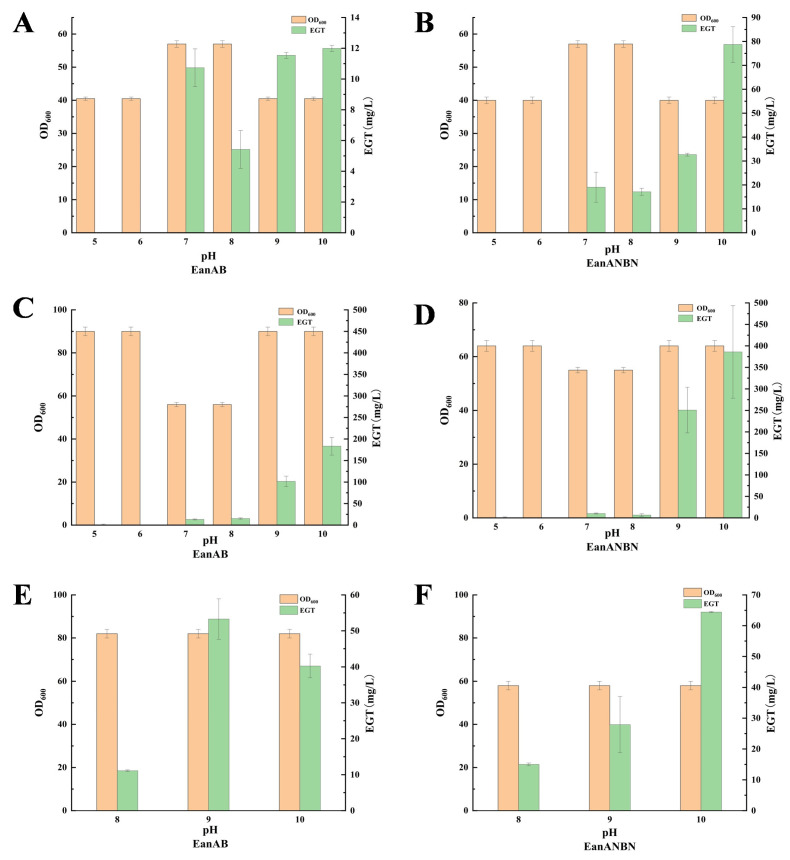
Effect of different initial pH at 25, 30, and 37 degrees on ergothioneine conversion. Initial OD and EGT yields of two recombinant bacteria at 25 degrees Celsius (**A**,**B**), initial OD and EGT yields of two recombinant bacteria at 30 degrees Celsius (**C**,**D**), and initial OD and EGT yields of two recombinant bacteria at 37 degrees Celsius (**E**,**F**) are shown.

Selected initial conversion temperatures were 25 °C and 30 °C, with six pH levels: 5, 6, 7, 8, 9, and 10. The results are shown in Figure 4. Both recombinant strains were unable to synthesize ergothioneine under acidic conditions, and overall, the EGT yield increased with rising pH levels, a trend more pronounced at 30 °C. At 30 °C, both recombinant strains achieved higher EGT yields compared to 25 °C, with the highest yields at an initial pH of 10, reaching (183.3 ± 7.8) mg/L and (386.06 ± 9.5) mg/L, respectively (Figure 4). Subsequently, the conversion at a higher temperature of 37 °C was investigated. It was found that the maximum EGT yield for both strains decreased to (54.8 ± 3.4) mg/L and (63.6 ± 0.2) mg/L compared to the conversion temperature of 30 °C (Figure 4). This decrease might be due to the increased carbon flux towards overflow metabolites such as lactic acid and 2,3-butanediol at higher temperatures [25].

### 3.5. Effect of Precursor Amino Acid Addition on EGT Yield

**Figure 5:**
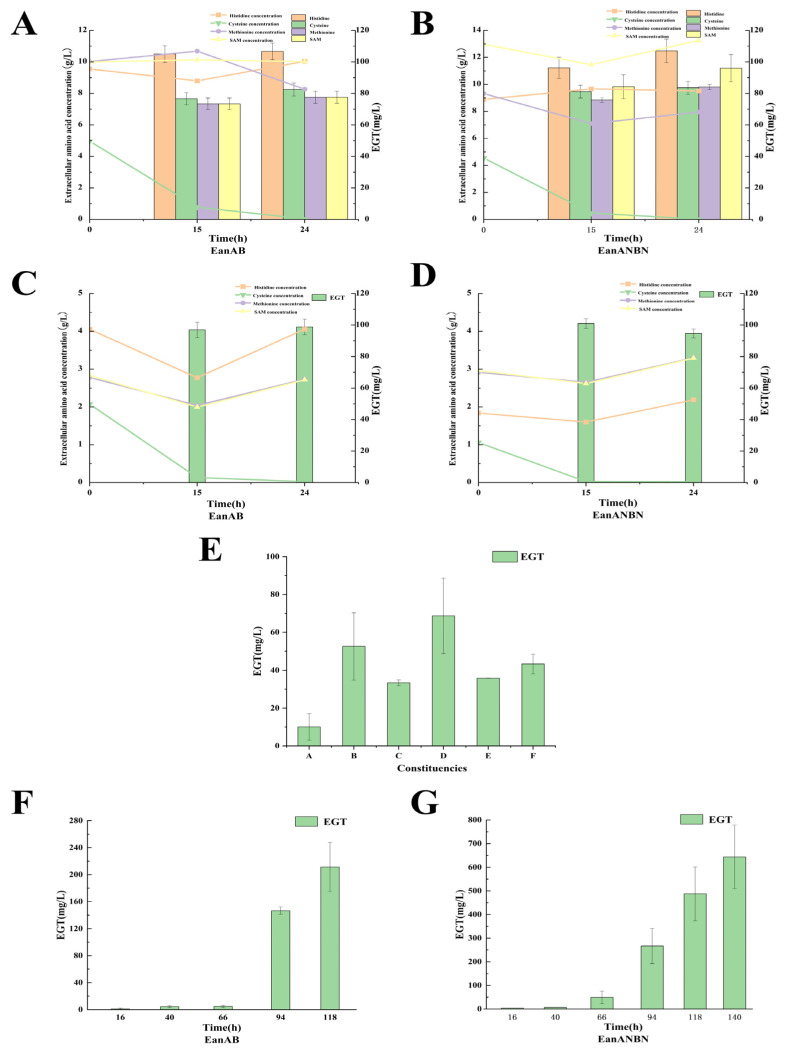
Yield of EGT by two recombinant bacteria under different conditions. Changes in extracellular amino acid concentration and EGT yield after exogenous addition of a single amino acid precursor (**A**,**B**), changes in extracellular amino acid concentration and EGT yield after exogenous addition of four mixed amino acid precursors (**C**,**D**), effect of the addition of different groups of precursor amino acids on EGT yield (**E**), and final yield of EGT in recombinant shake flask culture (**F**,**G**) are shown.

Ergothioneine is an amino acid derivative, and its synthesis in the *Chlorobium limicola* pathway involves three precursor amino acids [10,13]. Histidine provides the backbone for ergothioneine synthesis, methionine generates the active methyl donor S-adenosylmethionine, which provides the methyl group for ergothioneine synthesis under the action of methyltransferase, and cysteine provides the sulfur source for the sulfuration of the imidazole ring in histidine betaine [13]. Adding precursor amino acids to the medium during whole-cell transformation is a direct strategy to increase precursor supply [30]. To investigate the main precursor limitations for ergothioneine synthesis in the initial state, exogenous histidine, cysteine, methionine, and S-adenosylmethionine were added to the initial fermentation medium. The extracellular amino acid content and ergothioneine yield within 24 h were measured when adding single amino acids and mixed amino acids, as shown in Figure 5.

The recombinant strains EanAB and EanANBN showed the highest ergothioneine production when histidine was added for 24 h compared to the addition of the other three amino acids alone. The extracellular histidine concentration did not change significantly, suggesting a possible issue with histidine transport in *Bacillus licheniformis* [30]. Future studies could improve the ability to utilize histidine in *Bacillus licheniformis* so as to improve the generation of the intermediate metabolite HER. When all four amino acids were added together, the extracellular histidine concentration initially decreased and then increased, indicating that *B. licheniformis* has the ability to produce histidine. The measurement of extracellular amino acids revealed that cysteine was utilized most significantly, being almost completely consumed within 24 h. Cysteine serves as the sulfur source in the synthesis process, and its deficiency is a major limiting factor for ergothioneine production, making it essential to add cysteine during the transformation [13]. *B. licheniformis* showed poor absorption of methionine, resulting in the lowest ergothioneine yield when methionine was added alone (Figure 5). S-adenosylmethionine, as a hydrolysis product of methionine, can act as a methyl donor, and both methionine and S-adenosylmethionine maintained stable extracellular levels [10,13,31]. Therefore, different combinations of these two precursor amino acids were tested to determine the optimal combination.

### 3.6. Optimization of Precursor Amino Acid Composition to Enhance EGT Production

Based on the results above, it was established that the addition of histidine and cysteine is necessary when supplementing with exogenous amino acids. Various amino acid combinations were compared to the control group without amino acid addition (Table 3). It was found that adding 10 g/L each of histidine, methionine, and cysteine resulted in the highest ergothioneine yield of 68.72 mg/L (Figure 5), which represents an increase of 583.78% compared to the control group without additional amino acids (ergothioneine yield of 10.05 mg/L). Therefore, this combination and concentration of amino acids were used for subsequent additions.

Finally, to verify the ergothioneine production of the two recombinant strains after optimizing transformation conditions and establishing precursor amino acid combinations and concentrations, both strains were cultured in a transformation buffer with a final concentration of 10 g/L each of histidine, methionine, and cysteine at 30 °C and an initial pH of 10.0. As shown in Figure 5, the recombinant strain EanAB achieved a maximum ergothioneine yield of (211.39 ± 36.4) mg/L after 118 h of fermentation, while the strain EanANBN reached a maximum yield of (643.8 ± 135) mg/L after 140 h of fermentation.

### 3.7. Commercial Feasibility and Scalability of Producing Ergothioneine by Bacillus licheniformis

In this study, we used *Bacillus licheniformis*, a GRAS strain, as a chassis strain to construct a *Chlorobium limicola* pathway for ergothioneine production, offering more possibilities for ergothioneine production. Currently, the commonly used chassis strain for ergothioneine production is *Escherichia coli*. We will explore the greater commercial feasibility of using *Bacillus licheniformis* over *Escherichia coli* for ergothioneine production through the following aspects.

#### 3.7.1. Growth Characteristics and Fermentation Conditions

*Bacillus licheniformis* has a broader growth temperature range, typically growing well between 15 °C and 60 °C [32]. In contrast, *Escherichia coli* has a relatively narrow optimal growth temperature, usually around 37 °C [33,34,35]. This enables *Bacillus licheniformis* to have less stringent requirements for temperature control during large-scale fermentation processes, allowing it to adapt to more diverse environmental conditions and reducing the precision requirements and energy consumption of temperature control equipment. For example, during fermentation production in regions with significant natural environmental temperature fluctuations, *Bacillus licheniformis* is more likely to maintain a stable growth state and ergothioneine synthesis ability [32,36,37].

*Bacillus licheniformis* exhibits a higher tolerance to dissolved oxygen and can maintain a certain level of metabolic activity under relatively low dissolved oxygen concentrations. In large-scale fermenters, this is beneficial for reducing aeration costs and agitation intensity, thereby lowering equipment investment and operating costs [38]. In comparison, *Escherichia coli* has relatively strict requirements for dissolved oxygen and requires higher aeration rates and more intense agitation to maintain sufficient dissolved oxygen levels. During large-scale production, this will increase the complexity of equipment and energy consumption [39,40].

#### 3.7.2. Advantages in the Scale-Up Process

*Bacillus licheniformis* is less likely to produce large amounts of viscous substances during the fermentation process. Therefore, during large-scale fermentation, the rheological properties of the fermentation broth are relatively stable, which is conducive to mass transfer and heat transfer processes. It can reduce dead zones and local concentration non-uniformity within the fermenter, making the fermentation process more stable and efficient and easier to scale up [41,42,43]. However, *Escherichia coli* may produce more extracellular polysaccharides and other viscous substances under certain circumstances, resulting in an increase in the viscosity of the fermentation broth. This affects the transfer of nutrients and oxygen and increases the difficulties and risks during the scale-up process [44,45].

#### 3.7.3. Cost-Effectiveness

Raw Material Costs: *Bacillus licheniformis* can utilize a wider range of carbon and nitrogen sources, including some inexpensive industrial and agricultural wastes, such as starch residues, molasses, and corn steep liquor. These wastes are widely available and have low costs. In some cases, the resource utilization of these wastes can even be achieved, thereby significantly reducing the cost of the culture medium [46]. In contrast, *Escherichia coli* has relatively strict requirements for the components of the culture medium and usually requires high-purity sugars and amino acids as carbon and nitrogen sources, resulting in relatively higher costs [47,48].

Downstream Processing Costs: The proportion of ergothioneine produced by *Bacillus licheniformis* that is secreted extracellularly is relatively high, which simplifies the subsequent separation and purification processes and reduces complex and costly steps such as cell disruption [49,50]. In comparison, most of the ergothioneine produced by *Escherichia coli* accumulates inside the cells and requires cell disruption for extraction, increasing the complexity and costs of downstream processing, including aspects such as equipment investment, energy consumption, and the use of chemical reagents [51,52].

#### 3.7.4. Sustainability

Environmental Impact: Due to its relatively strong environmental adaptability and extensive substrate utilization ability, *Bacillus licheniformis* generates relatively fewer wastes during the production process, resulting in less pollution pressure on the environment. Meanwhile, its low demand for dissolved oxygen and relatively low aeration and agitation requirements also reduce the environmental burden caused by gas emissions and energy consumption [46,53,54]. During large-scale fermentation, *Escherichia coli*, due to its strict growth conditions and high aeration and agitation requirements, will generate more carbon dioxide emissions and energy consumption, having a relatively greater impact on the environment [55,56].

#### 3.7.5. Resource Utilization and Circular Economy

The ability of *Bacillus licheniformis* to utilize industrial and agricultural wastes as raw materials for the culture medium helps to promote the development of the circular economy. For example, converting the wastes generated during the starch processing process into nutrient sources for ergothioneine production realizes the reuse of wastes, reduces the dependence on new resources, and improves the resource utilization efficiency of the entire production process [46,57,58]. In contrast, *Escherichia coli*’s dependence on high-quality culture medium raw materials limits its potential in terms of resource recycling [59].

In conclusion, compared with *Escherichia coli*, the use of *Bacillus licheniformis* for ergothioneine production has obvious advantages in terms of scalability, cost-effectiveness, and sustainability, demonstrating higher commercial feasibility. It is expected to become a superior choice for future industrial production of ergothioneine. However, further optimization of production processes and technological parameters is still required to fully exploit its advantages and achieve large-scale, efficient, and sustainable ergothioneine production.

## 4. Conclusions

This study focused on the production of ergothioneine (EGT) by utilizing genetically engineered *Bacillus licheniformis*. The ergothioneine synthase genes, namely EanA and EanB from *Chlorbium limicola*, the methyltransferase EanAN from *Bacteroidales* bacterium, and the sulfurtransferase EanBN from *Anaerobacillus alkalidiazotrophicus*, were cloned and then expressed in Bacillus licheniformis. Subsequently, the recombinant strains EanAB and EanANBN were constructed. Through whole-cell conversion and the optimization of factors such as temperature, initial pH value, and precursor amino acid addition, the recombinant strain EanANBN achieved the highest yield of ergothioneine at (643.8 ± 135) mg/L.

Compared with *Escherichia coli*, *Bacillus licheniformis* demonstrates greater commercial feasibility and advantages in terms of scalability, cost-effectiveness, and sustainability. It has a broader growth temperature range, higher tolerance to dissolved oxygen, is less likely to generate viscous substances during the fermentation process, and is capable of utilizing a wider variety of carbon and nitrogen sources. Moreover, it simplifies the downstream processing procedures, has a lesser impact on the environment, and contributes to the promotion of the circular economy. Nevertheless, further optimization of the production process and technological parameters is still required to fully exploit its potential and achieve large-scale and efficient production of ergothioneine, making it a promising candidate for the future industrial production of this compound.

## Figures and Tables

**Table 1:** Plasmids and strains used in this study.

Names	Description	Sources
Plasmids		
pHY300-PLK	Expression vetor, TetR	Lab stock
pHY-P2-EanA	pHY300-PLK containing EanA	This work
pHY-P2-EanB	pHY300-PLK containing EanB	This work
pHY-P2-EanAN	pHY300-PLK containing EanAN	This work
pHY-P2-EanAN	pHY300-PLK containing EanBN	This work
pHY-P2-EanAB	pHY300-PLK containing EanA, EanB	This work
pHY-P2-EanANBN	pHY300-PLK containing EanAN, EanBN	This work
Strains		
*B. licheniformis*	Wild-type (ATCC 9945a)	Lab stock
*E. coli* JM109	*E. coli* Wild-type	Lab stock
*B. licheniformis*-PHY	*B. licheniformis* (ATCC 9945a) harboring pHY300-PLK	This work
*E. coli*-EanAB	*E. coli* JM109 harboring pHY-P2-EanAB	This work
*E. coli*-EanANBN	*E. coli* JM109 harboring pHY-P2-EanANBN	This work
EanAB	*B. licheniformis* (ATCC 9945a) harboring pHY-P2-EanAB	This work
EanANBN	*B. licheniformis* (ATCC 9945a) harboring pHY-P2-EanANBN	This work

**Table 3:** Different combinations and concentrations of amino acids are added exogenously.

	Final Amino Acid Concentration					
Transformation Buffer	No Added Amino Acids	Histidine, Methionine, Cysteine			Histidine, Cysteine, SAM	Histidine, Methionine, Cysteine, SAM
Constituencies	A1, A2	B1, B2	C1, C2	D1, D2	E1, E2	F1, F2
Concentration	0 g/L	2.5 g/L	5 g/L	10 g/L	10 g/L, SAM 5 g/L	10 g/L, SAM 5 g/L

## Data Availability

The original contributions presented in this study are included in the article. Further inquiries can be directed to the corresponding authors.
